# Quercetin, a Flavonoid with Great Pharmacological Capacity

**DOI:** 10.3390/molecules29051000

**Published:** 2024-02-25

**Authors:** Eber Josue Carrillo-Martinez, Flor Yohana Flores-Hernández, Adriana María Salazar-Montes, Hector Fabián Nario-Chaidez, Luis Daniel Hernández-Ortega

**Affiliations:** 1 Unidad de Biotecnología Médica y Farmacéutica, Centro de Investigación y Asistencia en Tecnología y Diseño del Estado de Jalisco, Guadalajara 44270, Mexico; 2 Instituto de Investigación en Enfermedades Crónico-Degenerativas, Centro de Universitario de Ciencias de la Salud, Universidad de Guadalajara, Sierra Mojada 950, Guadalajara 44340, Mexico; 3 Sistemas y Servicios Oncológicos de Latinoamérica S.A. de C.V., Zapopan 45079, Mexico; 4 Centro de Investigación Multidisciplinaria en Salud, Centro Universitario de Tonalá, Universidad de Guadalajara, Tonalá 45425, Mexico

**Keywords:** quercetin, antioxidant, free radicals, pharmacological properties, nanoparticles

## Abstract

Quercetin is a flavonoid with a low molecular weight that belongs to the human diet’s phenolic phytochemicals and nonenergy constituents. Quercetin has a potent antioxidant capacity, being able to capture reactive oxygen species (ROS), reactive nitrogen species (RNS), and reactive chlorine species (ROC), which act as reducing agents by chelating transition-metal ions. Its structure has five functional hydroxyl groups, which work as electron donors and are responsible for capturing free radicals. In addition to its antioxidant capacity, different pharmacological properties of quercetin have been described, such as carcinostatic properties; antiviral, antihypertensive, and anti-inflammatory properties; the ability to protect low-density lipoprotein (LDL) oxidation, and the ability to inhibit angiogenesis; these are developed in this review.

## 1. Introduction

The human body actively balances the production of free radicals (FRs) and antioxidants, ensuring that oxygen (O_2_) toxicity only manifests in pathological states. In this context, antioxidants, comprising a diverse array of substances such as vitamins, minerals, natural pigments, other plant compounds, and enzymes, can thwart the detrimental effects of free radicals [1]. Natural compounds are frequently associated with health and well-being benefits, driving research into the properties and applications of polyphenols. Polyphenols are natural compounds exclusively synthesized by plants, primarily existing in the form of glucosides and akin to phenolic compounds. These molecules are predominantly found in fruits, vegetables, green tea, and whole grains.

On the other hand, the basic structures of flavonoids are aglycones (the sugar-free fragment of the corresponding glycoside), and flavonoids are commonly found in glycosidic form. All flavonoids share the fundamental diphenyl propane structure (C6-C3-C6), where the phenolic rings (ring A and ring B) are typically connected by a heterocyclic ring. This heterocyclic ring (ring C) is often a closed pyran [2]. In addition, flavonoids possess a powerful antioxidant action, being able to scavenge a wide range of reactive oxygen species (ROS), reactive nitrogen species (RNS), and reactive chlorine species (RCS), which act as reducing agents by chelating transition-metal ions [3]. In particular, quercetin (3, 5, 7, 3′, 4′-pentahydroxy flavon) is a flavanol of the flavonoid polyphenol group [4]. The most common form of quercetin is rutin, which is usually glycosylated, formed by the addition of a glycoside group (a sugar, such as glucose, rhamnose, or rutin) instead of an -OH group (usually at the 3-position).

On the other hand, the sugar-free structure of quercetin is called aglycone, which is yellow in color and insoluble in cold water but has good solubility in alcohol and oil. However, a glycoside group increases water solubility compared to quercetin’s aglycone structure [5]. Like other polyphenols, quercetin has been reported to have bioactive properties such as antioxidant, antiaging, and anti-inflammatory effects. The antioxidant effect is achieved through the scavenging of free radicals and maintenance of the oxidative balance [6]. Its anti-inflammatory and antiallergic effects are mainly realized through its inhibition of lipoxygenase and cyclooxygenase (COX) [7]. Due to its antioxidant and other biological activities, quercetin has been widely used to treat various types of cancer, diabetes, obesity, nerve damage, and other diseases [8].

## 2. Quercetin Particularities 

### 2.1. Sources

Quercetin is the most widely distributed flavonoid in the world; it is usually found as a component in certain types of plants such as capers, tea, tomatoes, garlic, spinach, and brucella cabbage; in fruits such as grapes, apples, berries, pears, and cherries; and in seeds, nuts, flowers, bark, and leaves. In addition, it is also found in certain plants of medicinal use such as *Hypericum perforatum* (St. John’s wort) and *Sambucus canadensis* (elderberry). Some foods contain large amounts of this compound, as listed in Table 1; for example, buckwheat contains up to 23 mg of quercetin per 100 g, and bee pollen contains almost 21 mg per 100 g. Most of the quercetin consumed daily in the diet is in the glycosidic form, which is formed by the addition of a glycosyl group (a sugar such as glucose, rhamnose, or rutin) instead of an -OH group (position 3), while the aglycosidic form is found in much smaller amounts. One of the significant sources of quercetin aglycone is the onion; quercetin aglycone is more abundant in an onion’s outermost layers, while the innermost layers contain more substantial quantities of quercetin glycosides. Up to 10% of an onion’s dry weight can comprise quercetin in various forms [5]. In addition, the polyphenolic profile of fruits and vegetables depends on the plant species, growing conditions, harvesting conditions, and storage methods. For example, storage at high or very low temperatures reduces the quercetin content in foods. Price, in 1997, evaluated the curing process of onions at 28 °C for six months, during which he observed a decrease in quercetin content between 50 and 60%, while when immersed in water at 100 °C, only 25–33% of their content decreased. In contrast, the quercetin content in strawberries stored at 20 °C for nine months showed an increase of about 32% [9,10].

### 2.2. Physicochemical Properties 

**Figure 1:**
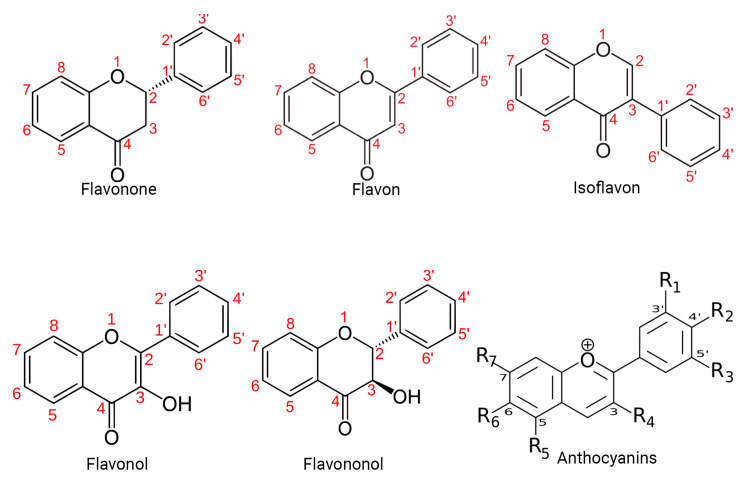
Structural diversity of flavonoids. This figure presents a visual representation of the structural diversity of flavonoid molecules. Variations in the hydroxylation pattern and oxidation state of the central pyran ring lead to the formation of a wide spectrum of compounds (Eber J. Ca. Mtz. 2023. ChemDraw^®^).

Specifically, polyphenols can be broadly classified into flavonoids and nonflavonoids. On the other hand, the structural diversity of flavonoid molecules arises from variations in the hydroxylation pattern and oxidation state of the central pyran ring, resulting in a broad spectrum of compounds, including flavanols, anthocyanidins, anthocyanins, isoflavones, flavones, flavonols, flavanones, and flavanonols (some of these structures are depicted in Figure 1) [11].

**Figure 2:**
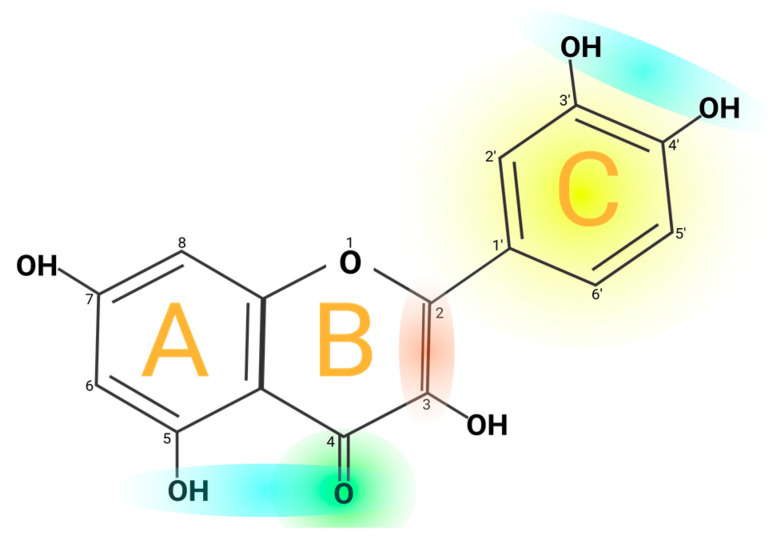
Chemical structure of quercetin. The figure shows the chemical characteristics that give it its antioxidant capacity, the most important of which is the catechol or dehydroxylated B-ring structure (yellow). Other important characteristics are the presence of unsaturation in the C-ring (red) and the presence of a 4-oxo in the C-ring (green); the oxygen in position 4 and the hydroxyl in position 5 allow it the capacity to chelate ions such as Cu^++^ and Fe^++^ (blue). On the other hand, in total, quercetin has 5 functional hydroxyl groups with the potential to be conjugated and that differ in their chemical reactivity following the order of reactivity 3 > 7 > 3′ > 4′ >> 5. The phenolic hydroxyl groups of quercetin act as donors of electrons, and these are responsible for the capture activity of free radicals, in particular the catechol structure with 2 hydroxyl groups in the neighboring positions, which is notably superior to other arrangements in electron donation (Eber J. Ca. Mtz. 2023. ChemDraw^®^).

Quercetin’s structure is 2-(3,4-dihydroxyphenyl)-3,5,7-trihydroxy-4H-chromen-4-one with a molecular weight of 302.24 g/mol and a melting point of 316 °C, and its molecular formula is C_15_H_10_O_7_. Quercetin consists of five hydroxyl groups (Figure 2), which determine the biological activity of the compound [2,12]. Naturally, quercetin is distributed in the form of derivatives, either in glycosidic form, mainly attached to the C-3 carbon with glucose, rhamnose, and rutinose, or linked to others, and it is very rarely distributed as an aglycone [13]. On the other hand, quercetin in its aglycone form is a bright lemon-yellow powder, insoluble in water but soluble in alcohol and lipids. In addition, the aglycone has been reported to have a minor effect in vivo and poor bioavailability in human plasma after oral intake of quercetin [14,15]. 

However, quercetin is highly lipophilic in nature due to the presence of five hydroxyl groups. However, the solubility of quercetin derivatives depends on the type of substituent molecules present in the OH group. The O-methyl, C-methyl, and prenyl derivatives of quercetin are lipophilic and are widely found on the surface of leaves, flowers, and fruits of the Labiatae or Compositae families [16]. Nevertheless, the glycosylation of quercetin increases its hydrophilic properties, and these glycosylated derivatives are soluble in the cytosol of plants, are easily transported to all parts of plants, and are mainly stored in the vacuoles [4]. In addition, the unique chemical structure of quercetin is responsible for its potent antioxidant properties. The structural groups responsible for quercetin’s stability and antioxidant activity are the ortho-dihydroxy or catechol group and the 3- and 5-OH groups in conjugation with the 4-oxo group [17]. Therefore, quercetin donates a proton to free radicals such as 2,2-diphenyl-1-picrylhydrazyl (DPPH) and is converted to quinone intermediates that are stabilized by the electrons donated by these functional groups. Quercetin derivatives, such as C3 and C4OH glycoside derivatives, have a lower H^+^ donating capacity. In addition, the reducing potential of the C3OH derivatives of quercetin is higher compared to its aglycone form [18,19].

### 2.3. Bioavailability and Pharmacokinetics

Bioavailability is the fraction of an ingested substance absorbed and available at the place of action. It is known that the bioavailability of quercetin is usually relatively low (0.17–7 μg/mL), less than 10% of what is consumed, due to its poor water solubility (hydrophobicity), chemical stability, and absorption profile. The bioavailability of quercetin depends on its chemical structure, origin, physicochemical properties, and whether it is consumed in parallel with other compounds, e.g., fats and pectin. In addition, a characteristic feature of the bioavailability of these metabolites is their slow elimination since their half-life ranges from 11 to 48 h, which could favor their accumulation in plasma after repeated intakes [20]. Several studies suggest that glycosidic quercetin is metabolized in the gastrointestinal tissue and liver before it enters the blood and is distributed to the internal organs. The acidic environment in the stomach does not facilitate the absorption of quercetin bound to sugars. However, this process is mediated by gastrointestinal enzymes such as phlorizin lactase hydrolase and cytosolic β-glucosidases in the colon, microbial β-glucosidases, and α-rhamnosidase [21].

Furthermore, quercetin cannot be absorbed by a passive mechanism because it is too polar to cross the phospholipid bilayers in the cell walls of the epithelium. However, thanks to metabolic enzymes secreted by the human body and the intestinal microbiota, quercetin undergoes chemical transformations that allow it to be absorbed in the gastrointestinal tract [22]. Concerning quercetin aglycone, which is highly lipophilic, it is proposed that it could cross the enterocyte membrane by simple diffusion [4]. On the other hand, in tissue, quercetin and quercetin glycosides from foods appear conjugated, either glucuronidated, sulfated, or methylated, suggesting that the in vivo bioactivity of quercetin may be due to its metabolites [21]. Quercetin glycosides can be absorbed by an active transport mechanism due to sodium-dependent glucose transporter 1 (SLGT1), a protein embedded in the cell walls of the gastrointestinal epithelium. Ingested quercetin glycosides can also be absorbed due to the activity of lactase-phlorizin hydrolase (LPH), an enzyme that removes the sugar moiety, resulting in a more hydrophobic quercetin aglycone. After absorption, the aglycone form can passively diffuse into the hepatic portal vein and then be transported throughout the body. In the intestine, biotransformation of quercetin results in the production of methyl metabolites, sulfate, and glucuronide, which tend to be more readily absorbed and can be measured in urine and blood to determine bioavailability [22,23]. 

To improve their bioavailability, several formulation strategies have been developed, such as various encapsulation techniques, in which an active ingredient is enclosed in another material to enhance its handling, dispersibility, stability, and/or functionality, including nanoencapsulation; emulsification; the use of liposomes or hydrogels; and complexation with other molecules such as cyclodextrin and cocrystallization. However, studies that promote the achievement of high bioavailability, whether in vivo or ideally in clinical intervention studies, still need to be strengthened [23]. Most published studies examined quercetin and/or its metabolites in the urine and plasma of relatively small numbers of volunteers, where there is less variation in metabolites derived from absorption in the small intestine compared to catabolites derived from the action of the colonic microbiota. Thus, dietary history, genetic polymorphisms, and variations in intestinal microbiota metabolism have been found to play an essential role in the bioavailability of quercetin [24].

### 2.4. Human Absorption and Metabolism of Quercetin 

As mentioned, quercetin is in the form of glycosides in vegetables and fruits. The degree of absorption of quercetin is given by the glycosides’ nature and binding site at positions 3, 5, 7, or 4 [25,26]. For example, Ullah H. et al. determined the absorption of various forms of quercetin in human volunteers using healthy subjects with ileostomy to avoid bacterial degradation in the colon. Their results showed that quercetin absorption was 52% for an onion-rich meal, 24% for quercetin supplementation, and 17% for routine supplementation [25,27,28]. Once ingested, quercetin glycosides are rapidly hydrolyzed by the enzyme β-glucosidase in the epithelial cells of the upper small intestine, much of which is subsequently absorbed.

Similarly, the glucuronic acid in quercetin and its sulfuric acid derivatives are more readily absorbed than quercetin. However, it has not been reported whether quercetin and its derivatives are stable in gastric acid; therefore, there are no reports on whether they can be absorbed in the stomach [23]. In addition, purified quercetin glycosides can interact with sodium-dependent glucose transport receptors in the mucosal epithelium and, therefore, can be absorbed by the small intestine in vivo [29].

**Figure 3:**
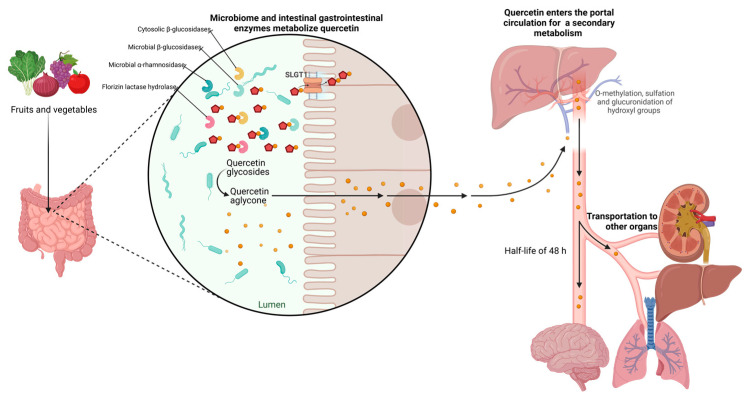
Metabolism of quercetin. The diagram illustrates the enzymatic transformations undergone by quercetin within the biological system. Key steps include deglycosylation by enterobacteria facilitating its absorption, and hydroxylation, glycosylation, and methylation processes, leading to the formation of various metabolites in the liver. The figure highlights the network of metabolic reactions involved in the biotransformation of quercetin, shedding light on its fate within the body (Eber J. Ca. Mtz. 2023. BioRender).

However, rutin and other quercetin glycosides bound to oligosaccharides or polysaccharides are absorbed in the lower part of the digestive tract (large intestine) in the form of aglycones through deglycosylation by enterobacteria (*Eubacterium ramulus*, *Clostridium orbiscindens*, *Eubacterium oxidoreducens*, *Butyrovibrio* spp.). In contrast, quercetin monoglucosides such as isoquercitrin and quercetin-4’-glucoside (Qu4’G) are absorbed in the upper part of the intestine (small intestine) after enzymatic hydrolysis by β-glucosidase and/or lactase-phlorizin hydrolase (LPH) in the intestinal mucosa derived from intestinal microbiota, as shown in Figure 3. Some of the quercetin monoglucosides can be taken up by sodium-glucose cotransporter 1 (SGLT-1). These metabolites enter the gastrointestinal tract via multidrug resistance-associated protein 2 (MRP2) and are subsequently transported through the blood vessels to the liver, where they are subjected to secondary metabolism [30]. After absorption, quercetin binds to albumin and is transported primarily to the liver via the portal vein and to other organs, including the small intestine, colon, and kidney. In the liver, it undergoes O-methylation, glucuronidation, and/or sulfation to form its conjugates quercetin-3-glucuronide, quercetin-30-sulfate, and isorhamnetin-3-glucuronide [31]. Conjugation reactions with glucuronic acid and/or sulfate appear to be the most common type of flavonoid metabolic pathway. For example, glucuronidation of flavanols occurs in human microsomes and the liver, and uridine diphosphate (UDP)- glucuronosyltransferase 1A9 (UGT-1A9) plays a vital role in this process. Kuhnle et al. reported that glucuronidation, O-methylation, and O-methyl-glucuronidation are part of flavonoid metabolism in the small intestine [32,33].

### 2.5. Quercetin Excretion 

Flavonoid glucuronides and sulfates are polar, water-soluble compounds that mammals efficiently excrete in urine and bile. When discharged in the bile, flavonoids pass into the duodenum and are metabolized by intestinal bacteria, producing cleavage products and/or the hydrolysis of glucoronoconjugates or sulfoconjugates [33]. The released metabolites can be reabsorbed and enter an enterohepatic cycle. The substitution in the flavonoid molecule, the degree of polarity, and the molecular weight determine the degree of biliary excretion [34]. It is worth mentioning that flavonoids are also eliminated through renal excretion in the liver after conjugation. Studies have demonstrated that the amount of excreted flavanols, as a proportion of intake, can vary from 0.8% to 1.4% depending on the dietary source of flavanols. The elimination half-life can also be influenced, with plasma levels of quercetin detected up to 48 h after consuming flavanols [5].

### 2.6. Pharmacological Properties 

In addition to its antioxidant capacity, different pharmacological properties associated with the administration of quercetin have been described, such as its carcinostatic properties (antimutagenic properties, suppression of cell proliferation, and induction of apoptosis), antiviral properties (against human immunodeficiency virus (HIV), hepatitis B virus (HBV), and herpes simplex virus (HSV)), antihypertensive properties (decrease in ventricular hypertrophy), anti-inflammatory properties, protection of low-density lipoprotein (LDL) from oxidation, and inhibition of angiogenesis [35]. Some of the main pharmacological properties of quercetin are described in more detail below.

#### 2.6.1. Antioxidant Properties

Oxidative stress refers to the pathophysiological responses caused by the excessive production of highly reactive molecules such as reactive oxygen species (ROS) in the body and the dysregulation of the oxidative–antioxidative balance when subjected to various harmful stimuli. Oxidative stress can damage mitochondrial DNA, denature intracellular proteins, cause lipid peroxidation, and drive inflammation [36]. Due to the phenolic hydroxyl group and a double bond, quercetin exhibits potential antioxidant activity. Quercetin is a potent scavenger of reactive oxygen species (ROS), protecting the organism against oxidative stress. Moreover, quercetin maintains oxidative balance, making it a robust antioxidant, and regulates the level of glutathione (GSH) in the body. Animal and cell studies have demonstrated that quercetin induces the synthesis of GSH. For instance, Xu and Dong described an increase in the expression of superoxide dismutase (SOD), catalase (CAT), and GSH with quercetin pretreatment [37]. In this regard, quercetin enhances the antioxidant capacity of the organism by elevating levels of glutathione (GSH). This is attributed to the fact that once oxygen free radicals are generated in the body, superoxide dismutase (SOD) rapidly captures O_2_^−^ and converts it into H_2_O_2_. Additionally, this enzyme catalyzes the breakdown of H_2_O_2_ into nontoxic H_2_O. This reaction necessitates GSH as a hydrogen donor [38].

In addition to its direct antioxidant properties inherent to the chemical family to which it belongs, quercetin can interact with the endogenous antioxidant network by activating the antioxidant responsive element (ARE). The ARE is a consensus sequence responsible for starting the transcription of most endogenous antioxidant enzymes, including notable ones such as NADPH dehydrogenase quinone 1 (NQO1), glutathione S-transferase (GSTA), thioredoxin (TRX), heme oxygenase (HMOX1), ferritin light chain (FTL), and inducible nitric oxide synthase (NOS). The mechanism by which quercetin accomplishes this process involves increasing the activity of the nuclear factor erythroid 2-related factor 2 (NRF2), enhancing its binding to the ARE, reducing its degradation, and consequently augmenting the synthesis of the involved enzymes [39]. 

#### 2.6.2. Cardiovascular Disease

##### Hypertensive Activity

High blood pressure is a condition in which the blood pressure in the arteries is constantly high. This condition can be dangerous because it can increase the risk of cardiovascular disease and stroke. Quercetin has been shown to benefit hypertension by lowering blood pressure in animal and human studies [40]. For example, Abdelghffar et al. found a decrease in systolic, diastolic, and mean blood pressure, a reduction in ventricular hypertrophy, and less damage to the renal and vascular parenchyma in a model of hypertensive rats. These findings are due in part to the vasodilator property of quercetin, which in turn is explained by quercetin’s ability to scavenge free radicals, which would usually activate the secretion of 8-iso-prostaglandin F2α, a potent vasoconstrictor hormone [41]. Likewise, a study published in the British Journal of Nutrition found that daily supplementation with quercetin (730 mg) for 12 weeks significantly reduced blood pressure in obese and overweight adults with prehypertension and stage 1 hypertension [42]. In addition, some underlying molecular mechanisms by which quercetin may be acting have been investigated. One theory is that quercetin causes endothelial nitric oxide synthase (eNOS) to be activated, producing nitric oxide (NO), a vasodilator that relaxes blood vessels and reduces blood pressure. According to several studies, quercetin increases eNOS activity and NO generation [43]. Another mechanism by which quercetin may exert its antihypertensive effect is the inhibition of angiotensin converting enzyme (ACE). Angiotensin II, a potent vasoconstrictor that raises blood pressure, is created by the ACE enzyme, and quercetin has been shown to inhibit ACE activity, reducing blood pressure [44]. 

##### Cardiovascular Protection 

Several studies have shown that quercetin has positive effects on cardiovascular diseases. Suri et al. evaluated the impact on vasoconstrictor and vasodilator reactions in the porcine heart, reporting that quercetin increased both cyclic guanosine mono-phosphate (cGMP) content and cGMP-dependent relaxations to glyceryl trinitrate (GTN) and sodium nitroprusside (SNP), as well as porcine receptor-mediated restrained contractions [45]. Also, a study with rats showed that quercetin (0.1–100 μM) relaxed the contraction induced by pretreatment with five mM norepinephrine in a concentration-dependent manner. It was concluded that quercetin induces Ca^++^ elevation, leading to NO production and activation of the endothelial cell’s Ca^++^-activated K^+^ channel (KCa) [46]. It should also be mentioned that quercetin showed anti-inflammatory properties in patients with coronary artery disease (CAD) through a decrease in nuclear factor kappa B (NF-Κb) transcriptional activity. Chekalina et al. determined the effect of quercetin in patients with CAD to test this effect. Eighty-five patients with CAD participated in the study. Thirty patients received quercetin at a daily dose of 120 mg for two months, while the remaining fifty-five patients considered as a control group received β-blockers, statins, and aspirin. Increased levels of interleukin 1β (IL-1β), tumor necrosis factor-α (TNF-α), and IL-10 were detected in the serum of CAD patients. Under the influence of quercetin, the levels of interleukin 10 (IL-10), IL-1β, and TNF-α were reduced. Also, quercetin decreased the expression of the βα kappa inhibitor (Ikβα) relative to the control group [47].

#### 2.6.3. Alzheimer’s Disease

It has also been proposed that quercetin may have therapeutic effects on Alzheimer’s disease (AD) through several molecular mechanisms. Oxidative stress and neuroinflammation play a crucial role in the pathogenesis of AD, and quercetin can scavenge free radicals and inhibit the production of inflammatory cytokines, thereby reducing oxidative stress and inflammation in the brain [48]. Another mechanism is quercetin’s ability to modulate the enzyme activity in clearing amyloid-beta (Aβ) plaques, a hallmark of AD pathology. Quercetin can increase the activity of neprilysin and insulin-degrading enzymes responsible for degrading amyloid beta (Aβ) plaques in the brain, thereby reducing their accumulation and toxicity [49]. Quercetin can also modulate the activity of kinases involved in tau protein phosphorylation, another hallmark of AD pathology.

Furthermore, quercetin can inhibit the activity of glycogen synthase kinase 3β, responsible for abnormal tau protein phosphorylation, thus reducing tau aggregation and neurofibrillary tangles in the brain [49]. Many in vivo studies demonstrated that the hydroxy-functional groups in the B-ring of quercetin are essential in inhibiting Aβ aggregation and altering mature fibrils by forming hydrogen bonds with the β-sheet structure. In addition, other studies have shown that quercetin increased the survival of neuronal cultures in vivo. Likewise, quercetin at 100 μM showed considerable inhibition of beta-site amyloid precursor protein cleaving enzyme 1 (BACE1) in an in vivo system by 11.85% [50]. The other characteristic pathological implication of AD is the appearance of neurofibrillary tangles (NFTs), with tau being the central protein. Several studies demonstrated that quercetin effectively inhibited tau accumulation through different mechanisms, such as a reduction in tau protein hyperphosphorylation, through the inhibition of glycogen synthase kinase 3β (GSK3β) activity [48].

#### 2.6.4. Antimicrobial Activity 

On the other hand, quercetin has been reported to exhibit antimicrobial activity against various microorganisms. Quercetin’s proposed mechanisms in microbial infections include inhibition of bacterial growth, biofilm formation, virulence factors, and modulation of the host immune response. Quercetin has been shown to exert antibacterial effects against Gram-negative and Gram-positive bacteria, including antibiotic-resistant strains such as methicillin-resistant staphylococcus aureus (MRSA) [51]. Recent studies have shown that quercetin can effectively alter the integrity of the bacterial cell membrane, thereby inhibiting bacterial growth. By transmission electron microscopy (TEM) analysis, Wang et al. observed that quercetin effectively altered the structural integrity of the cell wall and membrane of *E. coli* and *S. aureus*. In treated *E. coli*, the cell wall showed numerous structural abnormalities such as prominent lysis, cell distortion, leakage of cytoplasmic fluid, and cytoplasmic membrane separated from the cell wall. Similarly, in treated *S. aureus*, significant alteration of the cell wall, thinning of cell membranes, chromatin lysis, detachment of extracellular polysaccharides, and nuclear fragmentation were observed [51,52].

#### 2.6.5. Antiviral Activity 

Several studies have suggested that quercetin may have antiviral effects against a wide variety of viruses, including hepatitis C virus (HCV), influenza A virus (IAV), Chikungunya virus (CHIKV), and severe acute respiratory syndrome coronavirus 2 (SARS-CoV-2). Inhibition of viral replication, host cell entry, and host immune response are some of the mechanisms hypothesized for the antiviral effect of quercetin. The main molecular mechanisms of quercetin’s antiviral effects are inhibition of viral neuraminidase, proteases, deoxyribonucleic acid (DNA)/ribonucleic acid (RNA) polymerases, and modification of several viral proteins. It has also been documented to suppress HCV by binding to and inactivating the viral NS3 protease [14]. Rahman, M.A. et al. performed molecular docking in which they found that the significant interconnected nodes in the protein–protein network were protein kinase B (AKT1) (serine/threonine protein kinase), proto-oncogene tyrosine protein kinase sarcoma (SRC), epidermal growth factor receptor (EGFR), matrix metalloprotein (MMP9), kinase insert domain receptor (KDR), MMP2, insulin-like growth factor 1 receptor (IGF1R), protein tyrosine kinase 2 (PTK2), breast cancer resistance protein (BCRP), ATP-binding cassette super-family G (ABCG2), and mesenchymal–epithelial transition (MET) [53]. Quercetin inhibits viral retrotranscriptase, as is the case with Rauscher murine leukemia virus (RMLV), human immunodeficiency virus (HIV), and hepatitis B virus (HBV) [54,55]. Some published articles suggest that quercetin’s antiviral activity is due in part to a nonspecific protein denaturation mechanism that results in viral inactivation; however, some others offer the possibility that quercetin may bind to the surface receptors that viruses use to enter cells, thus blocking their infective capacity [56]. 

#### 2.6.6. Hepatoprotective Activity

Another essential aspect of quercetin is its hepatoprotective function. This function has been demonstrated in several animal models, specifically in models of chronic damage. Chronic liver diseases, in general, progress in a similar way; they start with a process of oxidative stress in the organ caused by a foreign agent that can be toxic to a virus, which in turn leads to an inflammatory state, which activates the processes of fibrogenesis. If the damage persists, then the disease will progress to what is known as liver cirrhosis characterized mainly by thick bundles of extracellular matrix, regeneration nodules, and necrosis [57].

#### 2.6.7. Oxidative Stress

Generally, at this stage, large amounts of free radicals (ROS such as superoxide anion (-O_2_), hydroxyl radical (-OH), peroxyl radical (R O_2_^−^), and alkoxyl radical (RO-) as well as RNS and ROC) are generated, which attack cell membranes, activating Kupffer cells and hepatocytes. The administration of quercetin has been shown to decrease the levels of oxidative stress associated with damage by sequestering and inactivating free radicals due to its antioxidant properties as well as by increasing the synthesis and activity of endogenous antioxidant enzymes [58].

#### 2.6.8. Inflammation

The inflammatory response is produced by infiltrating macrophages (inflammatory infiltrate) and liver-resident macrophages (Kupffer cells). It is characterized by the release of proinflammatory cytokines, such as interleukin-6 (IL-6) and tumor necrosis factor A (TNF-α), and profibrogenic cytokines, which in turn activate hepatic stellate cells (HSCs), the leading producers of the dense extracellular matrix. Experimental models have shown that quercetin can decrease the gene expression of these cytokines by downregulating their main transcriptional factor, nuclear factor κB (NFκB) [56].

#### 2.6.9. Fibrosis 

In the chronically damaged liver by any etiology, HSCs undergo a process of activation and transformation to a proliferative, fibrogenic, proinflammatory state and express α-smooth muscle actin (α-SMA) in their cytoplasm. Among the activators reported are transforming growth factor β-1 (TGF-β1), platelet-derived growth factor (PDGF), TNF-α, connective tissue growth factor (CTGF), endothelin-1, epithelial growth factor (EGF), fibroblast growth factor (FGF), and insulin-like growth factor (ILGF). In addition, it has been shown that the administration of quercetin significantly decreases the gene expression of some of these cytokines, including TGFβ1, which is reflected in a decrease in the amount of activated HSC and the amount of extracellular matrix and collagen deposits [59]. 

#### 2.6.10. Cirrhosis

Activated HSCs produce types I, III, and IV collagen, thus initiating the fibrosis process, where the normal cells of the organ are gradually replaced by extracellular matrix, leaving few functional hepatocytes as islets in the middle of a distorted tissue. It has been reported that quercetin, in addition to preventing the development of liver fibrosis, also participates in the activation of certain antifibrogenic factors (MMP2, MMP9, and TGFβ3), which leads to an improvement in the functional status of the organ and thus avoids the establishment of the pathology [60]. On the other hand, the antiproliferative effect of this compound on activated HSCs, characterized by uncontrolled proliferation, has been described. The use of quercetin allows the arrest of these cells during the growth phase 1 (G1) of the cell cycle through the increase in tumor suppressor proteins such as protein 53 (p53) and cell cycle inhibitors protein 21 (p21) and protein 27 (p27); it also suppresses the expression of cyclin D1, D2, E, and A and promotes apoptosis through the release of cytochrome C by increasing apoptosis antigen 1(FAS) and FAS ligand [61].

#### 2.6.11. Diabetes

There is evidence to suggest that quercetin may have beneficial effects on diabetes. Several mechanisms have been proposed for the antihyperglycemic action of quercetin, including increasing insulin sensitivity, promoting glycogen synthesis, and improving insulin resistance. In addition to that, quercetin promotes insulin sensitization by stimulating pancreatic β-cell proliferation, which improves glucose metabolism and insulin secretion [62]. It has also been reported that quercetin is an inhibitor of α-glucosidase and α-amylase. Furthermore, quercetin improved plasma insulin levels and lowered blood glucose in streptozotocin (STZ)-induced diabetes model by maintaining β-cells, thereby enhancing the effect of serum insulin [63]. In addition, quercetin has been shown to stimulate glucose uptake in isolated cells by promoting GLUT4 expression and endogenous GLUT4 translocation through upregulation of estrogen receptor-α, subsequently increasing the phosphorylation of the phosphatidylinositol-3-kinase/Akt (PI3K/Akt) signaling pathways [64].

#### 2.6.12. Arthritis

Arthritis is associated with decreased mobility, and its symptoms often include swelling, pain, stiffness, and redness. There are over 100 types of arthritis, the most common being osteoarthritis (OA), gout, and rheumatoid arthritis. Genetic, hormonal, and environmental factors have been linked to arthritis. Treatment of arthritis includes pharmacological steroids, nonsteroidal anti-inflammatory drugs (NSAIDs), and/or surgery [65]. On the one hand, quercetin reduces pain and inflammation associated with arthritis, as evidenced in a mouse study where it inhibited mechanical knee joint hyperalgesia, edema, and leukocyte recruitment. Mechanisms of quercetin included the inhibition of oxidative stress, production of cytokines such as cyclooxygenase-2 (COX-2) and proteoglycan degradation, and activation of the nuclear factor erythroid 2-related factor 2 (Nrf2)/heme oxygenase-1 (HO-1) (Nrf2/HO-1) signaling pathway [66]. Thus, Guo H. et al. [67] demonstrated that quercetin activated the P110α/AKT/mammalian target of rapamycin (p110α/AKT/mTOR) signaling pathway by targeting p110α, revealing its promising potential to delay the OA process by inhibiting cartilage ECM degradation and increasing chondrocyte proliferation. For this, a rat model was established to simulate osteoarthritis in vivo. It was concluded that quercetin exerts an anti-osteoarthritis effect by inhibiting MMP13 expression and increasing collagen deposition Ⅱ in vivo [67]. In addition, quercetin has been reported as a potential drug for treating rheumatoid arthritis, as quercetin administration alone was more effective than methotrexate in reducing joint inflammation in mice. Mechanisms included decreased levels of TNF-α, IL-1β, IL-17, and monocyte chemoattractant protein-1 (MCP-1) [68].

#### 2.6.13. Cancer 

Cancer remains a significant challenge worldwide, both in developed and developing countries. One possible avenue for cancer treatment is using plant secondary metabolites, which have shown promise in treating various diseases. Therefore, researchers are actively searching for new plant species and new compounds that can serve as effective treatments against multiple forms of cancer. Quercetin has been shown to have an antiproliferative and proapoptotic effect on various cell lines. Table 2 shows some examples; the concentrations used range from 7 nM to 100 µM, which could be used in humans since clinical studies indicate that plasma concentrations of up to 400 µM are well tolerated and are not associated with adverse effects [69]. On the other hand, its impact on the reduction of solid tumors has also been demonstrated in murine models in vivo, where patients subjected to the treatment showed significant improvements [70]. There are currently seven active clinical protocols involving quercetin to treat oncological conditions; three of them are still in the recruitment phase, while three more protocols have already completed the recruitment phase and are under development. Finally, there is one fully completed protocol, but the results of this study are not yet available; the study involves the use of sulindac and quercetin in the prevention of colon cancer [71].

**Figure 4:**
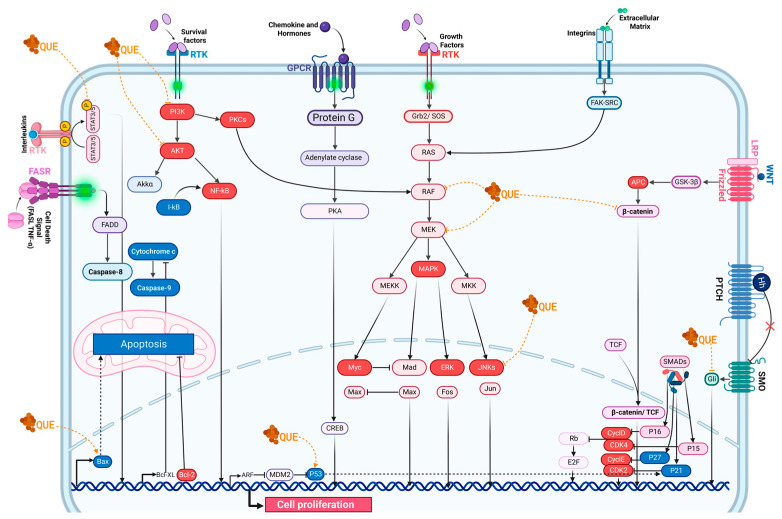
Effect of quercetin on the main signaling pathways involved in cancer. The figure shows some of the main signaling pathways involved in cancer. The increase in the activity of some of them results in an increase in cell proliferation, while the increase in some others increases cell apoptosis. In red are shown those molecules on which quercetin exerts an inhibitory effect, while in blue are those whose concentration and activity are increased when cells are exposed to quercetin (Eber J. Ca. Mtz. 2023. Bio-Render).

The carcinostatic effect of quercetin can be explained in part by its interaction with some of the main intracellular signaling pathways involved in cancer, as shown in Figure 4, which activate the transcription of proteins necessary for cell cycle progression and are therefore considered potential therapeutic targets: LY294002, a chemical compound, and PI3-kinase inhibitor, which has been modeled on the structure of quercetin and binds to the ATP-binding site of the enzyme in several different orientations, are currently available. Apparently, the number of -OH groups in the B-ring and the degree of C2–C3 instauration are essential determinants of quercetin’s particular bioactivity [72].

Another pathway susceptible to quercetin action is the protein kinase C (PKC) pathway, which is also downregulated [73]; by blocking the diacylglycerol (DAG) precursor of PKC, inhibition of this pathway leads to blocking the formation of phosphatidylinositol (3,4,5)-trisphosphate, which activates the entry of extracellular calcium [74]. In vivo, studies with human leukemia 60 (HL-60) cells report that concentrations between 20 and 30 µM are sufficient to exert an inhibitory effect on cytosolic PKC activity and membrane tyrosine protein kinase (TPK) activity. Within the same study, it is noted that a concentration of 80 µM is sufficient to block the activity of phosphatidylinositol (4,5) bisphosphate [75]. On the other hand, it has been shown that quercetin induces the inactivation of the AKT protein, an antiapoptotic protein, by decreasing its phosphorylation and also directly inactivates procaspase 9, thus blocking cellular apoptosis [76]. In addition, exposure of cells to a concentration of 50 µM resulted in the blockade of the extracellular signal-regulated kinases (ERK1/2) pathway. It showed better effects than when higher doses (75–100 µM) were used, as these doses reduced the expression of proapoptotic factors such as Bcl-2-associated X protein (Bax) and caspases 3 and 9 [77].

On the other hand, studies in human leukemia cells K562 show that the action of quercetin is not only focused on the inhibition of cell proliferation but also is able to induce apoptosis at concentrations of 80 µM and also causes a downregulation of cellular myelocytomatosis (c-myc) and Kirsten RAt sarcoma (K-ras) oncogenes [78]. The proapoptotic effect of quercetin can be partly explained because the compound’s antioxidative effect changes entirely to a prooxidant effect at high concentrations, which induces selective cytotoxicity [79]. In a different study, the selectivity of these processes was proven since quercetin was able to decrease cell viability in human glioma cultures of the U-118 MG cell line as well as an increase in death by apoptosis and cell arrest at the G2 checkpoint of the cell cycle. On the other hand, when noncancerous cells are exposed to quercetin, it exerts cytoprotective effects; in hippocampal cell cultures, quercetin is protective against ischemic damage [80]. Quercetin also interacts directly with mitochondria by modulating the mitochondrial transition pore (mPT), which controls the release of cytochrome C, second mitochondria-derived activator of caspase/direct inhibitor of apoptosis-binding protein with low pI (SMAC/DIABLO), and the mPT, which possesses a benzodiazepine binding site where quercetin binds and influences the opening and release of cytochrome C [81].

##### Synergistic Effect

Synergistic effect against breast cancer

In this sense, the synergistic effect of quercetin in combination with different drugs has been studied. For example, Liu et al. [82] found that quercetin combined with doxorubicin can induce multinucleation of invasive tumor cells, downregulate P-glycoprotein (P-gp) expression, increase cell sensitivity to doxorubicin, and accelerate apoptosis of breast cancer cells. For example, the growth of the human breast cancer cell line Michigan Cancer Foundation-7 (MCF7) was reduced with the optimal molar ratio of quercetin and doxorubicin [83]. Furthermore, several studies have shown that resveratrol, quercetin, and catechin can effectively block the cell cycle and reduce cell proliferation in vivo. Quercetin together with carboxyamidotriazole can block the signal transduction pathway by inhibiting phosphatidylinositol (PI) and phosphatidylinositol phosphate (PIP) activities [8]. In addition, Gupta et al. [84] constructed an encapsulation vector of quercetin and vincristine, demonstrating that this system can improve the action time of the two drugs and effectively enhance the anticancer effect. A large number of experimental results have shown that quercetin has a synergistic effect in the treatment of breast cancer [85].

Synergistic effect against prostate cancer

In prostate cancer, the combination of quercetin and arctigenin inhibits prostate cancer cell proliferation by inhibiting the expression of the androgen receptor (AR) and phosphoinositide 3-kinase (PI3K)/Akt pathways and several oncogenic small RNAs (including microRNA-21, microRNA-19b, and microRNA-148a) [74]. The same is observed with the coadministration of quercetin with 2-methoxy estradiol, which increases apoptosis by downregulation of caspase-3 protein. Furthermore, compared to a single treatment, quercetin was used with 2-methoxy estradiol to reduce the level of phosphorylated Akt protein (pAkt), B-cell lymphoma 2 (Bcl-2), Bax, vascular endothelial growth factor (VEGF) protein, and mRNA expression [86]. Finally, cotreatment with epigallocatechin gallate (EGCG) inhibited catechol-O-methyltransferase (COMT) activity, decreasing COMT protein content and thereby arresting the cell cycle of PC-3 human prostate cancer cells [86]. In another study, the synergistic treatment of tamoxifen and quercetin was also able to inhibit prostate tumor formation by regulating angiogenesis [8].

Synergistic effect against leukemia

In this regard, the coadministration of doxorubicin and quercetin significantly increased superoxide dismutase (SOD) activity and decreased malondialdehyde (MDA) content in the heart of mice. Therefore, combining quercetin and doxorubicin may enhance the effect of doxorubicin in treating leukemia [87]. Consequently, quercetin combined with cytosine arabinoside has been shown to improve leukemia treatment by inhibiting the formation of human leukemic cell colonies [88]. Also, the synergistic administration of quercetin with resveratrol in the induction of caspase-3 activity was significant compared to the groups of quercetin administered alone [89]. Similarly, combining quercetin with menadione at appropriate concentrations effectively reduced cell viability and may have improved therapeutic efficacy against leukemia [90]. Similarly, P-gp expression in cells was significantly decreased by quercetin treatment in combination with doxorubicin, also increasing the expression of phosphorylated N-terminal C-Jun kinase and p38 mitogen-activated protein kinase in chronic myelogenous leukemia cells (K562/adriamycin (ADR) cells) [91].

Synergistic effect in other types of cancer

Something similar occurs with other types of cancer. It was shown that quercetin can increase cisplatin-induced apoptosis by 16.3% in Hep-2 human laryngeal carcinoma cells [92]. The cumulative effect of quercetin and epigallocatechin gallate (EGCG) was also found to suppress the Janus kinase/signal transducer and activator of transcription (JAK/STAT) survival signaling cascade in CCA human embryonal rhabdomyosarcoma cells [93]. Reciprocally, quercetin in combination with cisplatin and oxaliplatin could be used to overcome drug resistance in cancer cells [79]. On the other hand, quercetin increased sensitivity to cisplatin in human osteosarcoma cell line 143B by modulating the expression ratio of miR-217-Kras [94]. Also, coadministration of 2.5 μM of EGCG, genistein, and quercetin suppressed the cell proliferation of a prostate cancer cell line (CWR22Rv1) by controlling androgen receptor and NAD (P)H: quinone oxidoreductase 1 (NQO1) expression [93]. In another study with T98G glioma cells, Bądziul et al. investigated that imperatorin combinations induced apoptotic cell death through downregulation of heat shock protein 27 (Hsp27) and Hsp72 expressions [95]. Another study observed a synergistic effect of quercetin and doxorubicin (DOX), which arrested the cell cycle in G2/M in the human colon cancer cell line HT29 [85]. Mahbub, Maitre, Haywood-Small, Cross, and Jordan-Mahy [92] suggested that the cumulative use of cyclophosphamide and quercetin minimized toxicological symptoms and improved fatigue behavior in patients with advanced colon cancer. 

### 2.7. Molecular Pathways Targeted by Quercetin

#### 2.7.1. Cell Cycle

Cell cycle progression is vital for cell homeostasis, and the interaction between cyclins, cyclin-dependent kinases (CDKs), and CDK inhibitors (CDKIs) is necessary to ensure its orderly progression. Quercetin causes cell cycle arrest by influencing several target proteins such as p53, p21, p27, cyclin B, D, and cyclin-dependent kinases. Thus, quercetin preferentially triggers cell cycle arrest through induction of p73 and p21 and inhibition of cyclin B, at the level of both transcription and translation. It can also downregulate cyclin B1 and cyclin-dependent kinase-1 (CDK-1), which are required for the orderly progression through the G2/M phase of the cell cycle. However, quercetin-induced G1/S arrest is no less common and occurs through the induction of p21 and simultaneous phosphorylation of the retinoblastoma protein (RBP), which inhibits G1/S cell cycle progression by blocking E2F1 [96]. Depending on the cell type, quercetin can arrest cells even in the G1 phase. Therefore, it has been shown that the induction of the G1 cell cycle arrest by quercetin causes a decrease in cyclins D1/Cdk4 and E/Cdk2 and an increase in p21 in vascular smooth muscle cells [97]. In addition, quercetin is known to be a potent inhibitor of topoisomerase II (TopoII), a cell cycle-associated enzyme necessary for DNA replication and chromosome segregation and DNA double-strand breaks (DSBs) in the DNA–TopoII–flavonoid ternary complex, the so-called DNA cleavage complex [73].

#### 2.7.2. Apoptosis

Another aspect is apoptosis, represented by specific cellular events, including blebbing, loss of cell adhesion, cytoplasmic shrinkage, DNA fragmentation, and activation of caspases through extrinsic and/or intrinsic pathways. Evidence suggests that quercetin can induce apoptosis (cell death) through caspase-3 and caspase-9 activation, cytochrome c release, and poly ADP ribose polymerase (PARP) cleavage [98]. Quercetin has been described to induce apoptosis in several human cell lines. It has also been suggested that quercetin induces the loss of mitochondrial membrane potential, leading to the activation of the caspase cascade and cleavage of PARP. Different studies have shown that quercetin exposure can increase the expression of proapoptotic proteins such as Bax and cytochrome complex (Cyt c), leading to the release and translocation of apoptosis-inducing factors from the mitochondria to the nucleus [99]. In this regard, quercetin has also been shown to activate the intrinsic apoptosis pathway (e.g., in MDA-MB-231 cells) through the Ca^++^-mediated dissipation of mitochondrial membrane potential (MMP) and activation of caspases 3, 8, and 9 [36]. Alternatively, quercetin can activate Bcl-2 family proteins, caspases, and the concomitant blockade of PI3K/Akt and ERK signaling [100].

In addition, quercetin has been found to modulate proteins, such as NF-kβ and cyclooxygenase 2 (Cox-2), by suppressing the antiapoptotic proteins B-cell lymphoma-extra-large (Bcl-xL) and Bcl-2, increasing the expression of proapoptotic Bax proteins. It also decreases the ratio of Bcl-xL to B-cell lymphoma-extra-small (Bcl-xs) and increases Bax translocation to the mitochondrial membrane in human prostate cancer cells [86]. On the other hand, inhibition of signaling pathways, such as PI-3-kinase, Akt, and ERKs, and crosstalk between PI-3-kinase and ERKs was observed in quercetin-treated liver carcinoma cells. In addition, quercetin has been shown to activate Jun N-terminal kinases (JNKs) and increase JNK-dependent Bax and p53 expression in normal bronchial epithelial cells. Thus, evidence suggests that the apoptotic effects of quercetin may result from the inhibition of HSP kinases, followed by the downregulation of HSP-70 and HSP-90 protein expression [101]. 

#### 2.7.3. Wnt/β-Catenin Signaling

Moreover, the wingless and Int-1(Wnt/β)-catenin/T cell factor (TCF) signaling pathway is essential for cancer development and cellular processes such as growth and apoptosis. Quercetin has also been found to induce apoptosis in several cell types by inhibiting this signaling pathway, blocking the binding of β-catenin to Tcf-4 proteins, slowing the translocation of these proteins to the nucleus, and reducing β-catenin/TCF transcriptional activity [102]. In addition, quercetin has been found to inhibit the nuclear translocation of β-catenin in triple-negative breast cancer (TNBC), which lacks specific receptors. Coupled with this, quercetin can inhibit the epithelial–mesenchymal transition (EMT) and stimulate the mesenchymal–epithelial transition (MET) in TNBC; this is due to its ability to induce E-cadherin and suppress vimentin levels. Finally, quercetin has been shown to inhibit the nuclear accumulation of β-catenin and expression of β-catenin-regulated genes such as cyclin D1 and c-Myc, which may prevent the metastatic behavior of TNBC and lung cancer through Snail-mediated EMT [103,104].

#### 2.7.4. p53 Activity

Recent studies suggest that quercetin can selectively induce apoptosis in cancer cells by targeting the activity of p53, a tumor suppressor protein that plays a crucial role in maintaining cellular integrity and preventing tumor formation. Loss of p53 function is frequently observed in several types of cancer, making it an attractive target for cancer therapy [105]. Quercetin has been shown to stimulate the acetylation and phosphorylation of p53 in cancer cells [106]. Due to the increased p53-mediated transcription of proapoptotic genes such as Bax, P53-upregulated modulator of apoptosis (PUMA), and phorbol-12-myristate-13-acetate-induced protein 1 (NOXA), cancer cells eventually undergo apoptosis. In addition, mouse double minute 2 (MDM2), an onco-protein that promotes p53 destruction, can be inhibited by quercetin [107]. Alternatively, p53 contributes to quercetin-induced NAG-1 (nonsteroidal anti-inflammatory drug-activated gene-1) expression, leading to apoptosis (e.g., in HCT116 colon cancer cells). In addition to p53, several other transcription factors (such as early growth response 1 (EGR-1), specificity protein 1 (Sp1), and peroxisome proliferator-activated receptor gamma (PPARγ)) are involved in quercetin-induced NAG1 expression [108].

#### 2.7.5. Ras Expression

Quercetin has been shown to act in the Ras cell signaling pathway, a crucial signaling system that controls cell proliferation, differentiation, and survival. Many human malignant tumors share the common feature of dysregulation of the Ras pathway. According to several studies, quercetin can prevent Ras proteins from being expressed. In one study, quercetin was found to inhibit the expression of Harvey rat sarcoma (H-Ras), K-Ras, and neuroblastoma rat sarcoma (N-Ras) in human breast cancer cells, leading to decreased proliferation and increased apoptosis [109]. For instance, using several human cancer cell models, quercetin was shown to preferentially reduce the oncogenic Ras protein over wild-type Ras by a post-translational mechanism. These findings indicate for the first time that quercetin reduced oncogenic Ras protein levels by accelerating proteasome-function-mediated degradation [110]. Cháirez Ramírez et al. stated that quercetin reduced the steady-state levels of p21-ras proteins in both colon cancer cell lines and primary colorectal tumors; these findings were confirmed by Western blot analysis and flow cytometry, which showed that p21-ras expression was reduced by approximately 50% of the control values. Quercetin was equally effective in inhibiting the expression of K-, H-, and N-Ras proteins. Furthermore, the effect of quercetin on Ras oncogene expression appeared to be specific and not merely a consequence of the general inhibition of protein synthesis [110].

#### 2.7.6. PI3K Signaling Pathway

Another aspect is the PI3K (phosphatidylinositol 3-kinase) signaling pathway, a lipid kinase that phosphorylates the 3’ position of the inositol ring of phosphatidylinositol. It plays a crucial role in cell growth, proliferation, differentiation, motility, survival, and intracellular trafficking. Quercetin is classified as a broad-spectrum inhibitor against PI3K [51]. Quercetin has been reported to inhibit PI3K/AKT activity and block proliferation in human leukemic cell lines [74]. In particular, quercetin reduced the PI3K/AKT/mTOR pathway and concomitantly decreased AKT phosphorylation at Ser473, suggesting that it also interfered with the kinase activity of the mTORC2 complex [110].

#### 2.7.7. NF-κB Signaling Pathway

On the other hand, the results of Chen et al. [111] indicate that quercetin could suppress TNF-α-induced apoptosis and inflammation by blocking the NF-κB signaling pathway in human umbilical vein endothelial cells (HUVECs), which could be one of the underlying mechanisms in the treatment of coronary disease [111]. Also, studies suggest that it fights inflammation by suppressing the inhibitor of nuclear factor ΚB (IκB) phosphorylation, NF-κB translocation, activating protein-1 (AP-1), and reporter gene transcription [47]. In that sense, quercetin is a potential inhibitor of NF-κB, which adds to its growth inhibitory and/or chemo-preventive properties. Specifically, it suppresses the development of H460 lung cancer cells by inhibiting NF-κB and activating death receptors and cell cycle inhibitors [96]. Cheng, Huang, JH, Wu, and Cheng [35] also demonstrated that quercetin inhibited signaling pathways related to the inflammatory process, including phosphorylation of mitogen-activated protein kinases (MAPKs), IκBα/β kinase, c-Jun, cyclic adenosine monophosphate (cAMP) response element-binding protein (CREB), activating transcription factor 2 (ATF2), and nuclear factor (NF)-κB p65, and blocked the translocation of NF-κB p65 to the nucleus [35]. In addition to this, Sul and Ra [112] investigated the effects of quercetin on oxidative stress and inflammation in A549 lung epithelial cells by suppressing the nuclear translocation of nuclear factor kappa B (NF-κB) and reducing the levels of inflammatory cytokines, such as tumor necrosis factor (TNF)-α, interleukin (IL)-1, and IL-6 [112]. 

#### 2.7.8. Autophagy 

Another critical aspect is autophagy, a cellular process often associated with cancer cells’ survival advantage when subjected to internal and external stress. However, when autophagy is overstimulated, it can lead to a nonapoptotic cell death known as autophagy-induced cell death (type II programmed cell death (PCD)). In many cancer cell lines, quercetin has been shown to be a potent inducer of autophagy. This is achieved by inhibiting proteasomal activity and mTOR signaling. Different studies have demonstrated that quercetin inhibits proteasomal activity and blocks mTOR/eukaryotic translation initiation factor 4E-binding protein 1 (4EBP1)/P70 ribosomal S6 kinase (p70S6K) activity [96,113].

At the molecular level, Akt-mTOR signaling and hypoxia-induced factor 1α (HIF-1α) signaling can modulate quercetin-induced apoptosis. Quercetin has been shown to inhibit cell proliferation and induce autophagy in U87 and U251 glioma cells. Furthermore, quercetin promotes TRAIL-induced apoptosis in human lung cancer cells by activating autophagy flux, degrading p62, and increasing microtubule-associated protein 1 light chain 3B (LC3B) conversion. This raises the possibility of using quercetin in combination with TRAIL as a combined chemo-preventive strategy for lung cancer [96].

### 2.8. Nanoparticles as Therapy

The term “nano” derives from the Greek root meaning “dwarf”, representing a scale corresponding to one billionth of a meter (10–9 m). It is pertinent to distinguish between two concomitant disciplines: nanoscience and nanotechnology. Nanoscience encompasses the research and analysis of structures in the nanometer-scale range, i.e., those between 1 and 100 nanometers. On the other hand, nanotechnology refers to the practical application of knowledge derived from nanoscience in creating devices and other technological advances [114].

Nanotechnology represents the only viable platform for exploring new characteristics of matter and establishing collaborations with disciplines such as medicine, chemistry, biology, pharmaceuticals, optics, and even engineering. In recent decades, science and technology have addressed challenges in the health field, thus promoting a more efficient diagnostic and therapeutic system [114]. It is relevant to note that the concept of “nanotechnology” was initially coined by the American physicist and Nobel Prize winner Richard Feynman in 1959 during the annual meeting of the American Physical Society at the conference “There’s Plenty of Room at the Bottom” at the California Institute of Technology (Caltech) [115].

Despite its wide range of pharmacological activities, quercetin has properties that remain an obstacle to its in vivo application, such as its poor water solubility, weak bioavailability, oxidant instability, and intense confined biotransformation. In addition, another challenge in developing new drugs is how to transport the drugs to their target sites, thus reducing the amount of substances administered and, consequently, minimizing their side effects while improving their therapeutic efficacy [116].

It is a well-established fact that the large surface area offered by particle size reduction can significantly improve the dissolution rate and bioavailability according to the classical Noyes–Whitney equation [117]. For example, nanoparticles have demonstrated powerful advantages in delivering hydrophobic drugs such as quercetin, including high encapsulation efficiency, prolonged circulation time, tumor-specific biodistribution, controlled release, and enhanced therapeutic efficacy. Hence, numerous engineered nanoparticle platforms have been developed, and significant advances have been made in delivering quercetin to treat different diseases. Since then, liposomes, polymeric micelles, poly lactic glycolic acid (PLGA) nanoparticles, metal–organic frameworks, inorganic nanoparticles, biomacromolecule-based nanoparticles, and other nanoparticles have been developed to deliver quercetin and improve its pharmacological efficacy [118].

#### 2.8.1. Liposomes 

Liposomes can be described as nanocarriers that mimic cellular phospholipid bilayers. The phospholipids used to produce liposomes have hydrophilic and hydrophobic portions that allow the spontaneous formation of spherical lipid bilayers in aqueous formulations. The type of lipids chosen influences the properties of the liposomes, such as charge, size, and stiffness [119]. Patel et al. combined mycophenolic acid (MPA) with quercetin using liposomal nanoparticles (LNPs) to impede the metabolic rate of MPA and enhance the water solubility of quercetin. LNPs showed enhanced cellular uptake and cytotoxic effects due to clathrin- and caveolae-mediated endocytosis, resulting in a higher apoptosis rate than free drugs, their combinations, or individual liposomal formulations [120]. To evaluate the targeting ability, a liposomal drug delivery system was designed for the coadministration of doxorubicin (DOX) and quercetin, which enhanced drug accumulation and demonstrated enhanced cytotoxicity in MCF-7/ADR cells by downregulating P-glycoprotein (P-gp) without affecting adenosine triphosphate (ATP) production. In addition, in vivo antitumor studies showed that the liposomal drug delivery system (DOX/quercetin) inhibited MCF-7/ADR solid tumors and reduced P-gp overexpression without noticeable histological changes in the heart [121]. Shaji et al. used multilamellar vesicles (MLVs) with phosphatidylcholine and cholesterol in a 9:1 ratio to encapsulate quercetin, showing hepatoprotective activity in rats [122]. Thinking about tumor therapy, Yuan et al. worked with a tumor-bearing mouse model using lecithin/cholesterol/polyethylene glycol 4000 (PEG4000)/quercetin in a ratio of 13:4:1:6 and showed inhibition of tumor growth [123]. Long et al. used pegylated liposomes composed of lecithin and cholesterol to encapsulate quercetin, showing antitumor and antiangiogenic properties in mouse models of ovarian cancer [124].

#### 2.8.2. Lipid Nanoparticles

Lipid nanoparticles can be classified into nanostructured lipid carriers (NLCs) and solid lipid nanoparticles (SLNs). The latter comprises one or more solid lipids, which form a solid matrix, being an excellent vehicle for drug delivery due to their physical stability, protection against degradation, controlled release, and low cytotoxicity. However, their lipid matrix can undergo recrystallization during storage and prematurely release the encapsulated compound. In this context, NLCs have been developed to overcome this problem by mixing liquid lipids with solids, creating an imperfect structure with more cavities and the capacity to encapsulate drugs and avoid the premature release of the encapsulated compounds [122]. Several lipid nanoparticle approaches have been developed to increase the bioavailability of quercetin and target specific sites. Li et al. produced SLNs with glyceryl monostearate and soybean lecithin, recording increased gastrointestinal absorption of quercetin in rats [125].

On the other hand, Chen-yu et al. used glyceryl monostearate, stearic acid, and medium-chain triglycerides to prepare NLCs for topical administration in rats with ear edema. The results showed a suppression of edema in the animals [126]. Speaking a bit about cancer, in a study by Sun et al., it was possible to induce apoptosis of MCF-7 and MDA-MB-231 breast cancer cells using NLCs composed of 2.7% quercetin, 9.4% soy lecithin, 23.6% glycerol 1,3-dido-decanoate 2-decanoate, 6.7% glyceryl tripalmitate, 13.4% vitamin E acetate, and 44.2% Kolliphor^®^ HS 15 [127]. In addition, taking advantage of its neuroprotective properties, Dhawan et al. encapsulated quercetin in SLNs composed of Compritol 888 ATO and preadolescent 80 [128]. In another study, they tested this formulation in rats chronically administered with aluminum chloride, which causes the oxidative stress responsible for brain damage. The results showed that quercetin-loaded SLNs improved memory retention in rats with aluminum-induced dementia compared to quercetin alone and empty nanoparticles, indicating that this nanosystem may efficiently target the brain [122].

#### 2.8.3. Polymeric Nanoparticles 

Polymeric nanoparticles are formed by biodegradable polymers, which offer multiple advantages, such as being stable in blood, biodegradable, nontoxic, nonthrombogenic, nonimmunogenic, and noninflammatory; in addition, they do not activate neutrophils, avoid the reticuloendothelial system, and apply to various molecules, such as drugs, proteins, peptides, or nucleic acids. The versatility of these nanoparticles is based on the ability to select the most suitable polymer for the desired application [37,129]. Encapsulation of hydrophobic antitumor drugs in PEG-poly lactide microcapsules (PEG-PLA) has been reported to increase their circulation time and accumulation in tumor sites. Therefore, PEG-PLA microcapsules (PEG-PLA-Qu) have been used to administer quercetin and enhance its antitumor efficacy [130]. On the other hand, the PEG-PLA-Qu system promoted apoptosis in MDA-MB-231 cells with a higher intracellular amount of quercetin than free quercetin. At the same time, in a breast cancer xenograft model, significant increases in inhibitory effects and apoptotic cells were observed in a group treated with PEG-PLA-Qu compared with free quercetin at the same dose [118]. In this regard, Gu et al. developed triphenylphosphine–quercetin/doxorubicin–PEG–monoclonal antibody (TQ/DOX-PEG-monoclonal antibody (mAb)) microcapsules to overcome multidrug resistance (MDR) by inducing mitochondrial damage and blocking ATP supply [131]. Triphenylphosphine quercetin conjugation effectively caused mitochondrial damage with ROS accumulation and depolarization of the mitochondrial membrane potential, leading to a significant decrease in ATP supply to ATP-binding cassette (ABC) transporters. Therefore, intracellular DOX accumulation was greatly enhanced, and growth was drastically inhibited in MCF-7/ADR cells treated with TQ/DOX-PEG-mAb. Furthermore, in a mouse model bearing a DOX-resistant breast tumor, TQ/DOX-PEG-mAb markedly retarded tumor growth without adverse systemic toxicity [132].

##### PLGA Nanoparticles

Poly (lactic-co-glycolic acid), a Food and Drug Administration (FDA)-approved synthetic pharmaceutical excipient, has attracted much attention in drug delivery due to its controlled and sustained release, prolonged circulation time, and selective delivery. PLGA nanoparticles have demonstrated their potential to deliver a variety of therapeutic agents, from small-molecule drugs to biomacromolecules [118,133]. Ersoz et al. synthesized and characterized quercetin-loaded PLGA nanoparticles (Qu1NPs, Qu2NPs, and Qu3NPs) with different sizes and encapsulation properties by a single-emulsion solvent evaporation method. The diameters of quercetin-loaded Qu1NPs, Qu2NPs, and Qu3NPs were determined to be 215.2 ± 6.2, 282.3 ± 7.9, and 584.5 ± 15.2 nm, and all of them could effectively inhibit C6 glioma cells. Moreover, treatment with Qu1NPs significantly reduced the level of MDA in C6 cells, decreasing the intracellular oxidative stress. These results revealed that smaller PLGA nanoparticles could enhance the internalization of quercetin, its antitumor efficacy, and its antioxidant capacity in C6 cells [134]. In addition, PLGA can be used as a shell to coat other nanoparticles to improve their mechanical strength, stability, and biocompatibility. For example, gold (Au)-NPs-Qu PLGA nanoparticles induced JAK2 suppression, autophagy, and apoptosis, which inhibited cervical cancer cell proliferation, invasion, and migration, promoting tumor suppression in vivo and in vivo [135].

#### 2.8.4. Inorganic Nanoparticles

The development of inorganic materials as nanocarriers has also been introduced in recent decades and has shown great potential in drug delivery and disease diagnosis. The most-used inorganic nanoparticles are silica nanoparticles, metal oxide, graphene oxide, and gold nanoparticles [136].

#### 2.8.5. Silica Nanoparticles

Silica nanoparticles are composed of silicon (46.83%) and oxygen (53.33%) and are an excellent choice as drug carriers due to their structure and favorable surface chemistry. In addition, silica nanoparticles have been shown to have significant advantages such as ease of synthesis, tunability of pore size, and good endocytic behavior. Among the various silica nanoparticles, mesoporous silica nanoparticles (MSNs) are promising porous materials with exceptional surface properties, with submicron-sized pores capable of loading multiple molecules [137]. For example, folate-conjugated, multifunctional pH-sensitive, multifunctional envelope-type mesoporous silica nanoparticles (folic acid (FA)–iron (FE)–Santa Barbara amorphous-15 (SBA-15)–QU) were developed to transport quercetin for the treatment of colon cancer. FA-FE-SBA15-Qu treatment triggered mitochondria-dependent apoptosis and initiated c-Jun N-terminal kinase (JNK)-mediated H2A histone family member X (H2AX) phosphorylation and p53 activation in HCT 116 cells [138]. On the other hand, Sapino et al. used aminopropyl functionalized MSNs for the topical administration of quercetin. The results showed that MSNs increased the penetration of quercetin into the skin and, at the same time, inhibited the proliferation of human melanoma cells [139].

#### 2.8.6. Magnetic Nanoparticles

Metal oxide nanoparticles have attracted significant attention as drug carriers due to their tunable size and shape, surface chemistry, and different oxidation states. Magnetic iron oxide (Fe_3_O_4_) nanoparticles are representative candidates for a multifunctional metal oxide nanocarrier whose magnetic properties allow them to be guided to the target site by applying an external magnetic field [140]. For example, magnetic nanoparticles hold great promise for cancer therapy because external magnetic fields can direct them to the tumor site, and they are increasingly being used in conjunction with magnetic resonance imaging (MRI), magnetic hyperthermia, and magnetic targeting in diagnosing tumors [122]. In a preliminary study, Barreto et al. synthesized Fe_3_O_4_ nanoparticles and showed that they had a controlled release time. Daglioglu designed a Fe_3_O_4_SiO_2_(FITC)BTN/QU/DOX nanocarrier system comprising magnetic (Fe_3_O_4_) and optical fluorescein isothiocyanate (FITC) contrast as a dual imaging probe, biotin (BTN) as a tumor-specific targeting ligand, doxorubicin as an antitumor drug model, and quercetin as a chemosensitizer to modulate tumor MDR in A549/DOX cells, revealing a significant increase in intracellular delivery and retention and a decrease in cell viability [141].

Similarly, gold nanoparticles are highly versatile and can be functionalized with various ligands and biocompatible molecules to achieve specific targeting. Surface functionalization of gold nanoparticles (AuNPs) is essential for biomedical applications targeting particular disease areas and to allow them to interact selectively with cells or biomolecules. Surface conjugation is generally achieved by adsorption of the ligand to the gold surface. In addition, long-term circulating nanoparticles (NPs) are desirable in systemic applications, such as passive focalization of tumors and inflammatory sites. Furthermore, gold nanoparticles are generally considered biocompatible and have a relatively safe track record in medical applications. They are also visible under imaging techniques, such as electron microscopy and absorption spectroscopy, which facilitates monitoring their distribution and behavior in the body [142]. 

Otherwise, gold–quercetin nanoparticle conjugations (AuNPs-Qu-5) inhibited EGFR and the PI3K/Akt/mTOR/GSK-2β pathway, accompanied by induced positive upregulation of Bax and caspase-3 and negative upregulation of Bcl-2, resulting in a significant increase in apoptosis and nuclear condensation compared with free quercetin. In addition, there was a substantial difference in EMT markers such as vimentin, N-cadherin, Snail, Slug, Twist, and E-cadherin protein expression in response to AuNPs-Qu-5, inhibiting the migration and invasion of MCF-7 and MDA-MB cells [143].

#### 2.8.7. Extracellular Vesicles

On the other hand, extracellular vesicles (EVs) are nanometer-sized lipid bilayer vesicles secreted by metabolically active cells with the potential for functional and structural modifications for organ-specific drug delivery. Compared to other delivery agents, extracellular vesicles offer several advantages, including biocompatibility; negligible systemic toxicity and adverse effects; enhanced biodistribution; and high transmission efficiency with a tendency to deliver biomolecules, including proteins, peptides, lipids, and nucleic acids [144]. Previously, extracellular vesicles showed promising potential to provide a wide range of drugs and biomolecules as carriers with significantly enhanced bioavailability and high transmission across the blood–brain barrier (BBB). One study demonstrated that quercetin-nanoparticle-loaded exosomes showed enhanced bioavailability with a therapeutic effect in Alzheimer’s disease by inhibiting cyclin-dependent kinase 5 (CDK5), facilitating tau protein phosphorylation [145]. In another study, the authors designed exosomes containing quercetin nanoparticles and monoclonal antibodies against growth-associated protein (GAP)43 and found a therapeutic effect on cerebral ischemia by decreasing reactive oxygen species (ROS) [146]. The authors of a recently published study evaluated the nutraceutical properties of extracellular vesicles containing quercetin and saponin. The manufactured extracellular vesicles were loaded with quercetin and saponins extracted from black bean (*Phaseolus vulgaris* L.) along with three other phytochemicals simultaneously to deliver to the target site or recipient cells. The study concluded that the extracellular vesicles loaded with nanoparticles had higher bioactivity than the phytochemicals used alone [147].

**Table 2:** Antiproliferative and proapoptotic effects of quercetin on various cell lines.

Malignant Cell Line	Reference
HepG2 hepatocellular carcinoma cells	Parvez, Al-Dosari, Arbab, Al-Rehaily and Abdelwahid [54]
PC-3, LNCaP, and DU-145 human prostate cancer cells	Sharma, Raut, Baruah and Sharma [86]
MDA-MB-231 and AU565 human breast cancer cells	Molani Gol and Kheirouri [127]
Caco-2, DLD1, HT-29, SW620, HKE-3, HCT-116, FHC, DKO-4, and HKE-3 human colon cancer cells	Aziz, Lotfy, Said, El Ashry, El Tamany, Soliman, Abu-Serie, Teleb, Yousuf, Dömling, Domingo and Barakat [107]
HGC-27, NUGC-2, MKN-7, and MKN-28 human gastric cancer cells	SDF
143B highly metastatic human osteosarcoma cell line	Doghish, Hegazy, Ismail, El-Mahdy, Elsakka, Elkhawaga, Elkady, Yehia, Abdelmaksoud and Mokhtar [94]
HPB-ALL chronic leukemia B-cells	Shi, Su, Cui, Yu, Du, and Han [87]
H460 lung cancer cell lines	Sul and Ra [112]
MNT1, M10, and M14 human melanoma cells	Córdoba-Moreno, Mendes, Markus and Fernandes [68]
A2780s and A2780cp ovarian cancer cell lines	Vafadar, Shabaninejad, Movahedpour, Fallahi, Taghavipour, Ghasemi, Akbari, Shafiee, Hajighadimi, Moradizarmehri, Razi, Savardashtaki and Mirzaei [124]
HeLa cervical cancer cell lines	Shorobi, Nisa, Saha, Chowdhury, Srisuphanunt, Hossain and Rahman [14]

## 3. Conclusions

Quercetin has experienced a remarkable increase in research and use in medicine in recent years thanks to its outstanding pharmacological properties. Its excellent antioxidant, anti-inflammatory, and anticarcinogenic activity has captured the scientific community’s attention, prompting numerous studies focused on exploring its potential health benefits. Despite these attributes, obstacles have been encountered, mainly with the bioavailability and solubility of quercetin, which limit its clinical efficacy.

This review highlights the physicochemical properties of quercetin, especially the A (phenolic) and B (flavanone) rings of quercetin’s chemical structure, which are conjugated aromatic rings containing double bonds that give them hydrophobic properties. These hydrophobic regions influence the low solubility of quercetin in water. This, in turn, affects its absorption and bioavailability in the body. These obstacles have prompted the exploration of innovative solutions, with nanotechnology emerging as a critical strategy to overcome these problems. One proposed strategy is using nanoparticles, which can improve the solubility and bioavailability of quercetin, offering the possibility of the more efficient and precise delivery of the compound. This approach addresses quercetin’s intrinsic challenges and promises to improve its therapeutic efficacy and reduce potential side effects.

We also discuss its potent pharmacological activity, which has been shown to influence some molecular pathways, such as PI3K/AKT/mTOR, Wnt/β-catenin, and NF-κB, involved in various chronic degenerative diseases, which it accomplishes through the harmful modulation of pathways that induce cell proliferation and the activation of those that induce apoptosis. These are the main mechanisms responsible for its pharmacological effects. So far, the results have demonstrated quercetin’s great pharmacological capacity in treating various types of pathology, but more clinical trials are needed to prove its effectiveness. Clinical trials would allow for establishing effective doses and identifying possible adverse effects of prolonged therapy.

In summary, the convergence between the pharmacological properties of quercetin and the therapeutic innovations of nanotechnology represents an exciting field of research that has the potential to transform medical treatment and offer more effective and personalized solutions in the future. 

## Figures and Tables

**Table 1:** Main natural sources of quercetin.

Food Source	Quercetin Content (mg/100 g)
FRUITS	
Apple with skin (*Malus domestica*)	4.42
Acerola (*Malpighia emarginata*)	4.74
Arctic bramble (*Rubus arcticus*)	9.1
Blueberry (*Vaccinium caesariense*)	7.67
Cranberry (*Vaccinium macrocarpon*)	14.84
Elderberry (*Sambucus* spp.)	26.77
Fig (*Ficus carica*)	5.47
Plum (*Prunus domestica*)	12.45
Sea buckthorn (*Hippophae rhamnoides*)	7.4
Wolfberry (*Lycium barbarum*)	13.6
Common juniper (*Juniperus communis*)	46.61
Prickly pear (*Opuntia* spp.)	4.86
VEGETABLE PRODUCTS	
Sowthistle (*Sonchus oleraceus*)	16
Arugula (*Eruca sativa*)	7.92
Sparrow grass (*Asparagus officinalis*)	13.98
Swiss chard (*Beta vulgaris*)	7.5
Green chicory (*Cichorium intybus*)	6.49
Coriander (*Coriandrum sativum*)	52.9
Golden poppy (*Eschscholzia californica*)	26.3
Drumstick tree (*Moringa oleifera*)	16.65
Fennel (*Foeniculum vulgare*)	48.80
Leaf cabbage (*Brassica oleracea*)	7.71
Red lettuce (*Lactuca sativa*)	7.61
Mustard greens (*Brassica juncea*)	8.8
Okra (*Abelmoschus esculentus*)	20.97
Onions (*Allium cepa*)	20.3
Perennial wall-rocket (*Diplotaxis tenuifolia*)	66.19
New Mexico chile (*Capsicum annuum*)	15
Sweet potato (*Ipomoea batatas*)	16.94
SPICES AND HERBS	
Caper bush (*Capparis spinosa*)	180.77
Dill (*Anethum graveolens*)	55.15
Oregano (*Origanum vulgare*)	7.3
Tarragon (*Artemisia dracunculus*)	11
Turmeric (*Curcuma longa*)	4.92
Buckwheat (*Fagopyrum esculentum*)	15.38

## Data Availability

Not applicable.

## Abbreviations

ROS	Reactive oxygen species
RNS	Reactive nitrogen species
RCS	Reactive chlorine species
LDL	Low-density lipoprotein
FR	Free radical
COX	Cyclooxygenase
SGLT1	Sodium glucose cotransporter 1
DPPH	2,2-diphenyl-1-picrylhydrazyl
LPH	Lactase-phlorizin hydrolase
Q4’G	Quercetin-4’-glucoside
MRP2	Multidrug resistance-associated protein 2
UDP	Uridine diphosphate
UGT-1A9	UDP-glucuronosyltransferase 1A9
HIV	Human immunodeficiency virus
HBV	Hepatitis B virus
HSV	Herpes simplex virus
GSH	Glutathione
SOD	Superoxide dismutase
CAT	Catalase
ARE	Antioxidant response element
NQO1	NAD (P)H dehydrogenase quinone 1
GSTA	Glutathione S-transferase
HMOX1	Heme oxygenase
FTL	Ferritin light chain
NOS	Nitric oxide synthase
NRF2	Nuclear factor erythroid 2-related factor 2
ENOS	Endothelial nitric oxide synthase
NO	Nitric oxide
ACE	Angiotensin-converting enzyme
cGMP	Cyclic guanosine monophosphate
GTN	Glyceryl trinitrate
SNP	Sodium nitroprusside
CAD	Coronary artery disease
NF-Κb	Nuclear factor kappa B
TNF-α	Necrosis tumoral alfa
IL-10	Interleukin 10
IL-1β	Interleukin 1β
Ikβα	Βα kappa inhibitor
AD	Alzheimer’s disease
Aβ	Amyloid-beta
BACE1	Beta-site amyloid precursor protein-cleaving enzyme 1
NFTs	Neurofibrillary tangles
GSK	Glycogen synthase kinase
MRSA	Methicillin-resistant staphylococcus aureus
TEM	Transmission electron microscopy
HCV	Hepatitis C virus
IAV	Influenza A virus
CHIKV	Chikungunya virus
SARS-CoV-2	Severe acute respiratory syndrome coronavirus 2
DNA	Deoxyribonucleic acid
RNA	Ribonucleic acid
NS3	Nonstructural protein 3
Akt	Serine/threonine protein kinase
SRC	Sarcoma
EGFR	Epidermal growth factor receptor
MMP	Matrix-metalloprotein
KDR	Kinase insert domain receptor
IGF1R	Insulin-like growth factor 1 receptor
PTK2	Protein tyrosine kinase 2
ABCG2	ATP-binding cassette super-family G
BCRP	Breast cancer resistance protein
MET	Mesenchymal–epithelial transition
RMLV	Rauscher murine leukemia virus
HSCs	Hematopoietic stem cells
α-SMA	Alpha smooth muscle actin
TGF-Β1	Transforming growth factor beta 1
PDGF	Platelet-derived growth factor
CTGF	Connective tissue growth factor
FGF	Fibroblast growth factor
EGF	Epithelial growth factor
ILGF	Insulin-like growth factor
TGFβ3	Transforming growth factor beta 3
G1	Growth 1
P53	Protein 53
P21	Protein 21
P27	Protein 27
FAS	Apoptosis antigen 1
STZ	Streptozotocin
GLUT4	Glucose transporter type 4
PI3K	Phosphoinositide 3-kinases
OA	Osteoarthritis
NSAIDs	Nonsteroidal anti-inflammatory drugs
COX-2	Cyclooxygenase-2
Nrf2	Nuclear factor erythroid 2-related factor 2
HO-1	Heme oxygenase-1
MTOR	Mammalian target of rapamycin
ECM	Cartilage extracellular matrix
MCP-1	Monocyte chemoattractant protein-1
PKC	Protein kinase C
DAG	Diacylglycerol
HL-60	Human leukemia 60
TPKs	Tyrosine protein kinases
ERK1	Extracellular signal-regulated kinase
BAX	Bcl-2-associated X-protein
C-MYC	Cellular myelocytomatosis
K-RAS	Kirsten rat sarcoma
MPT	Mitochondrial permeability transition
SMAC	Second mitochondria-derived activator of caspase
DIABLO	Direct inhibitor of apoptosis-binding protein with low pI
MCF7	Michigan Cancer Foundation-7
PI	Phosphatidylinositol
PIP	Phosphatidylinositol phosphate
AR	Androgen receptor
BCL-2	B-cell lymphoma 2
VEGF	Vascular endothelial growth factor
COMT	Catechol-O-methyltransferase
SOD	Superoxide dismutase
MDA	Malondialdehyde
ADR	Adriamycin
EGCG	Epigallocatechin gallate
JAK	Janus kinase
STAT	Signal transducer and activator of transcription
HSP	Heat shock protein
NQO1	NAD (P)H:quinone oxidoreductase 1
Dox	Doxorubicin
CDKs	Cyclin-dependent kinases
CDKIs	CDK inhibitors
PRB	Retinoblastoma protein
TOPOII	Type II topoisomerase
DSBs	DNA double-strand breaks
PARP	Poly ADP ribose polymerase
CYT C	Cytochrome complex
MMP	Mitochondrial membrane potential
COX	Cyclooxygenase
BCL-XL	B-cell lymphoma-extra-large
BCL-XS	B-cell lymphoma-extra-small
JNKs	Jun N-terminal kinases
WNT	Wingless and Int-1
TCF	T cell factor
TNBC	Triple-negative breast cancer
EMT	Epithelial–mesenchymal transition
PUMA	P53-upregulated modulator of apoptosis
NOXA	Phorbol-12-myristate-13-acetate-induced protein 1
MDM2	Mouse double minute 2
NAG-1	Nonsteroidal anti-inflammatory drug-activated gene-1
EGR-1	Early growth response 1
SP1	Specificity protein 1
PPARγ	Peroxisome proliferator-activated receptor gamma
H-RAS	Harvey rat sarcoma
N-RAS	Neuroblastoma rat sarcoma
SER	Serine
MTORC2	Mammalian target of rapamycin complex 2
HUVECs	Human umbilical vein endothelial cells
I-Κβ	Inhibitor of NF-Κβ
AP-1	Activating protein-1
MAPKs	Mitogen-activated protein kinases
CAMP	Cyclic adenosine monophosphate
CREB	Camp-response element-binding protein
ATF2	Activating transcription factor 2
PCD	Programmed cell death
4E-BP1	Eukaryotic translation initiation factor 4E-binding protein 1
P70S6K	P70 ribosomal S6 kinase
HIF-1α	Hypoxia inducible factor 1 subunit alpha
TRAIL	Tumor necrosis factor-related apoptosis-inducing ligand
LC3B	Microtubule-associated protein 1 light chain 3B
PLGA	Poly-lactic-co-glycolic acid
MPA	Mycophenolic acid
LNPs	Lipid nanoparticles
P-Gp	P-glycoprotein
MLVs	Multilamellar vesicles
ATP	Adenosine triphosphate
PLA	Poly-lactic acid
NLCs	Nanostructured lipid carriers
SLNs	Solid lipid nanoparticles
QU	Quercetin
TQ	Thymoquinone
PEG	Polyethylene glycol
Mab	Monoclonal antibody
MDR	Multidrug resistance
ABC	ATP-binding cassette
FDA	Food And Drug Administration
NPs	Nanoparticles
MSNS	Mesoporous silica nanoparticles
FA	Folic acid
SBA-15	Santa Barbara amorphous-15
H2AX	H2A histone family member X
MRI	Magnetic resonance imaging
Sio2	Silicon dioxide
BBB	Blood–brain barrier
BTN	Biotin
FITC	Fluorescein isothiocyanate
EVS	Extracellular vesicles
GAP	Growth-associated protein
